# High-Throughput Screening Reveals Alsterpaullone, 2-Cyanoethyl as a Potent *p27^Kip1^* Transcriptional Inhibitor

**DOI:** 10.1371/journal.pone.0091173

**Published:** 2014-03-19

**Authors:** Brandon J. Walters, Wenwei Lin, Shiyong Diao, Mark Brimble, Luigi I. Iconaru, Jennifer Dearman, Asli Goktug, Taosheng Chen, Jian Zuo

**Affiliations:** 1 Department of Developmental Neurobiology, St. Jude Children’s Research Hospital, Memphis, Tennessee, United States of America; 2 Department of Chemical Biology and Therapeutics, St. Jude Children’s Research Hospital, Memphis, Tennessee, United States of America; 3 University of Bath, Bath, United Kingdom; Florida State University, United States of America

## Abstract

p27^Kip1^ is a cell cycle inhibitor that prevents cyclin dependent kinase (CDK)/cyclin complexes from phosphorylating their targets. p27^Kip1^ is a known tumor suppressor, as the germline loss of p27^Kip1^ results in sporadic pituitary formation in aged rodents, and its presence in human cancers is indicative of a poor prognosis. In addition to its role in cancer, loss of p27^Kip1^ results in regenerative phenotypes in some tissues and maintenance of stem cell pluripotency, suggesting that p27^Kip1^ inhibitors could be beneficial for tissue regeneration. Because p27^Kip1^ is an intrinsically disordered protein, identifying direct inhibitors of the p27^Kip1^ protein is difficult. Therefore, we pursued a high-throughput screening strategy to identify novel p27^Kip1^ transcriptional inhibitors. We utilized a luciferase reporter plasmid driven by the *p27^Kip1^* promoter to transiently transfect HeLa cells and used cyclohexamide as a positive control for non-specific inhibition. We screened a “bioactive” library consisting of 8,904 (4,359 unique) compounds, of which 830 are Food and Drug Administration (FDA) approved. From this screen, we successfully identified 111 primary hits with inhibitory effect against the promoter of *p27^Kip1^*. These hits were further refined using a battery of secondary screens. Here we report four novel *p27^Kip1^* transcriptional inhibitors, and further demonstrate that our most potent hit compound (IC_50_ = 200 nM) Alsterpaullone 2-cyanoethyl, inhibits *p27^Kip1^* transcription by preventing FoxO3a from binding to the p27^Kip1^ promoter. This screen represents one of the first attempts to identify inhibitors of p27^Kip1^ and may prove useful for future tissue regeneration studies.

## Introduction

p27^Kip1^ (also known as Cdkn1B) is a member of the Cip/Kip family of cell cycle inhibitors which are characterized by their ability to bind and inhibit cyclin dependent kinases (CDK)/cyclin complexes, halting cell cycle progression in the G1 phase [1]. Loss of p27^Kip1^ has been associated with some forms of cancer in humans, and germline *p27^Kip1^* deletion in mice results in sporadic pituitary tumors at old ages [2]–[6]. Although mutations in *p27^Kip1^* are not usually causative of cancer, it is often dysregulated and associated with a poor prognosis [7], [8] if detected in cancer. Because of these observations, screening for compounds to antagonize p27^Kip1^ levels has not been the focus of previous studies. Despite this, recent experiments have cast a light on how p27^Kip1^ may antagonize stem cell pluripotency [9] and regenerative processes within certain tissue types, giving some impetus for the identification of small molecules which decrease the levels of p27^Kip1^. Specifically, loss of p27^Kip1^ has been associated with regenerative phenotypes in spinal cord injuries [10], hepatocyte transplantation [11], and in the inner ear [12]–[15]. The inner ear is perhaps the best characterized organ in terms of p27^Kip1^ and its link to regeneration. Within the inner ear lies the organ of Corti, the sensory epithelial sheet which contains the sensory hair cells and their supporting cells. It was observed that p27^Kip1^ initiates its expression during embryonic development coinciding with the exit of these cells from the cell cycle [16], [17], implying a pivotal role for p27^Kip1^ in these cells. In the postnatal mouse cochleae, removal of p27^Kip1^ from normally quiescent supporting cells forced these cells to re-enter the cell cycle [12], [13], [18] and loss of p27^Kip1^ preceded conversion of supporting cells to sensory hair cells *in vitro* [14]. Indeed, p27^Kip1^inhibition therapy has been proposed for hearing restoration in mammals [14], [18]. Similarly, multiple cell cycle inhibitors are upregulated in older cells [19], implying that a cocktail of cell cycle inhibitors, including p27^Kip1^, may need to be developed to force proliferation to occur in older quiescent tissues. These observations led us to pursue screening strategies for reduction of p27^Kip1^.

p27^Kip1^ belongs to a class of proteins called intrinsically disordered proteins (IDPs), which lack stable secondary and tertiary structure. IDPs represent extremely difficult targets for the development of small molecule inhibitors [20] since the protein has little structure. Moreover, efforts to promote cytoplasmic localization of p27^Kip1^ for degradation are also problematic. Once p27^Kip1^ is excluded from the nucleus, it regulates cell migration and cancer metastasis [21], [22]. Thus, control of p27^Kip1^ protein abundance and localization impede direct targeting of p27^Kip1^. Because of these observations, we decided to pursue a transcription-based approach to antagonize p27^Kip1^.

*p27^Kip1^* transcription is known to be regulated by the Forkhead box O (FoxO) family of transcription factors [23], the Sex determining region T-box 2 (Sox2) [12], and E2F1 transcription factors [24]. FoxO3a is a well-studied transcription factor which can be modulated by reversible acetylation. At the p27^Kip1^ locus, it has been demonstrated that acetylation of FoxO3a prevents it from binding to the *p27^Kip1^* promoter [25], and eventually results in the nuclear exclusion of FoxO3a. Thus, the balance between acetylation and deacetylation of FoxO3a is required for proper *p27^Kip1^* transcription.

In this study, we chose to design a luciferase based cell assay and screen for small molecules which antagonize *p27^Kip1^* transcription. After the assay was validated, we screened our “bioactive” library of 8,904 (4,359 unique, 830 FDA approved) compounds and obtained 111 primary hits which inhibit *p27^Kip1^* promoter activity. These initial hits were narrowed down to 4 hits though our intensive secondary screens, and we chose to focus on our most potent compound, Alsterpaullone, 2-cyanoethyl (A2CE), to understand how *p27^Kip1^* transcription was modulated by this compound. Surprisingly, we discovered that known inhibitors of Sirtuin 2 (Sirt2), a deacetylase, mimicked A2CE effect on p27^Kip1^ transcription implicating Sirt2 deacetylation for the inhibitory effect of A2CE on *p27^Kip1^* transcription inhibition. Since Sirtuin 2 removes acetyl groups and would promote FoxO3a binding to the *p27^Kip1^* promoter, we analyzed this interaction using chromatin immunoprecipitation (ChIP) followed by quantitative real time PCR, and discovered that addition of A2CE prevented FoxO3a from binding to the *p27^Kip1^* promoter.

In this study, we established our p27^Kip1^ screening assay and validated it by screening our “bioactive” library. Within this library, we discovered novel compounds that repress p27^Kip1^ transcription and mechanistically described how the most potent hit achieved this inhibition. In total, this screen represents a novel tool to address the repression of p27^Kip1^ and yields new compounds to achieve this inhibition.

## Experimental Procedures

### Ethics Statement

All animal work conducted during the course of this study was approved by the Institutional Animal Care and Use Committee at St. Jude Children’s Research Hospital and was performed accordingly to NIH guidelines.

### Cell Culture

HeLa and HEK-293 cells were obtained from ATCC (HeLa #CCL-2, HEK #CRL-1573). 3T3-J2 (Swiss) cells were a gift from Dr. Richard Schlegel [26]. All cell types were grown in DMEM supplemented with 10% FBS (Life Tech, #16000044), 1x penicillin/streptomycin mix (Life Tech, 15140148). Cells were grown in a tissue incubator at 37°C, 5% CO_2_, 95% relative humidity. Transfections were carried out using lipofectamine LTX transfection reagent (Life Tech, #15338100) following the manufacturers protocol.

### Plasmids

Our original luciferase vector was obtained from Dr. Sakai [27], the p27^Kip1^ promoter was excised, and cloned into BamH1 sites from pGL4.17 (Promega). Cloning was verified by both restriction enzyme digests and DNA sequencing. The non-specific plasmid (SV40-LacZ) was obtained from Addgene (#188116).

### Assay Optimization

The number of cells transfected, amount of lipofectamine transfection reagent, and plasmid DNA were all optimized for maximal endogenous luciferase activity prior to screening. We found that 2x10^6^ cells plated into a T75 flask (Corning, #3776) plate, followed by transfection utilizing 30 μL of transfection reagent and 5 μg of plasmid DNA gave optimal luciferase values.

### High Throughput Screening

HeLa cells transiently transfected with the p27^Kip1^-Luciferase plasmid were seeded into white, solid-bottom, tissue culture-treated, 384-well polystyrene plates (Corning, #8804BC) at a density of 5×10^3^ cells per well in 25 μl media. Compounds including those previously described (8,904 with 4,359 unique) [28], [29], cycloheximide (Sigma, #C7698) (positive control; non-specific inhibition), titrations of cycloheximide (1-to-2 dilutions from 200 μM to 6.1 nM) or DMSO (Fisher Scientific, D128-1) (negative control) were transferred with a V&P 384-well pintool at 30 nl/well to give a final compound concentration of 12 μM in each individual wells. The final cycloheximide concentration was 200 μM and the final DMSO concentration was 0.12%. The assay plates were then incubated overnight at 37°C, 5% CO_2_, 95% relative humidity followed by alamar blue cytotoxicity assay (Life Tech, #DAL1100) and luminescence luciferase reporter activity assay (Envision HTS microplate reader, PerkinElmer, Model 2102) with SteadyLite HTS reagent (PerkinElmer, #6016981) [30], [31]. Briefly, after overnight incubation, each well of the assay plates received diluted alamar blue reagent (1-to-12 dilution with DPBS (Life Tech, #14190) at 5 μl/well and incubated for an additional hour at 37°C, followed by 5 minute room temperature incubation to cool down. The assay plates were then read for fluorescent signals (excitation wavelength of 492 nm and emission wavelength of 590 nm) with an Envision plate reader. Next, SteadyLite HTS reagent (25 μl/well) was dispensed into each well followed by 20 minute room temperature incubation. The luminescence signals for individual wells were then measured with an Envision plate reader. Compound activity was normalized to that of 200 μM of cycloheximide (as 100% inhibition) and 0.12% DMSO (as 0% inhibition). Primary hits were compounds with luciferase inhibitory activity >50% and the activity difference between luciferase inhibition and alamar blue inhibition (%luciferase inhibition - %alamar blue inhibition) >20%. One hundred and eleven primary hits were further tested in a dose response analysis (10 compound concentrations, following a 3-fold dilution scheme with final concentration ranged from 56 μM to 2.8 nM, except for A2CE which ranged from 100 μM to 1 nM) in triplicate. Similarly, 200 μM of cycloheximide and 0.56% DMSO were included as positive and negative controls, respectively. The final DMSO concentration was 0.56% in the dose response assays, in order to achieve the highest compound concentration at 56 μM. The activity data for individual chemicals were fit into sigmoidal dose-response curves if applicable to derive IC_50_ values with GraphPad Prism 6.01.

### Chromatin Immunoprecipitation (ChIP)

ChIP was performed on 5x10^7^ HeLa treated for 1 day with 10 μm A2CE or DMSO using the simple ChIP kit from Cell signaling (#9003). ChIP’s were quantitated by realtime PCR (Eppendorf, Realplex^2^) using Syber Green (Biorad #170880). Results are displayed as percent enrichment compared to the input. Primers for the *p27^Kip1^* promoter are from [25] p27-FP: 5′-acacacacatcctggcaaag-3′; p27-RP: 5′-agtgtcccaaagaagcatgg-3′.

### LacZ Luminescence

**Figure 1:**
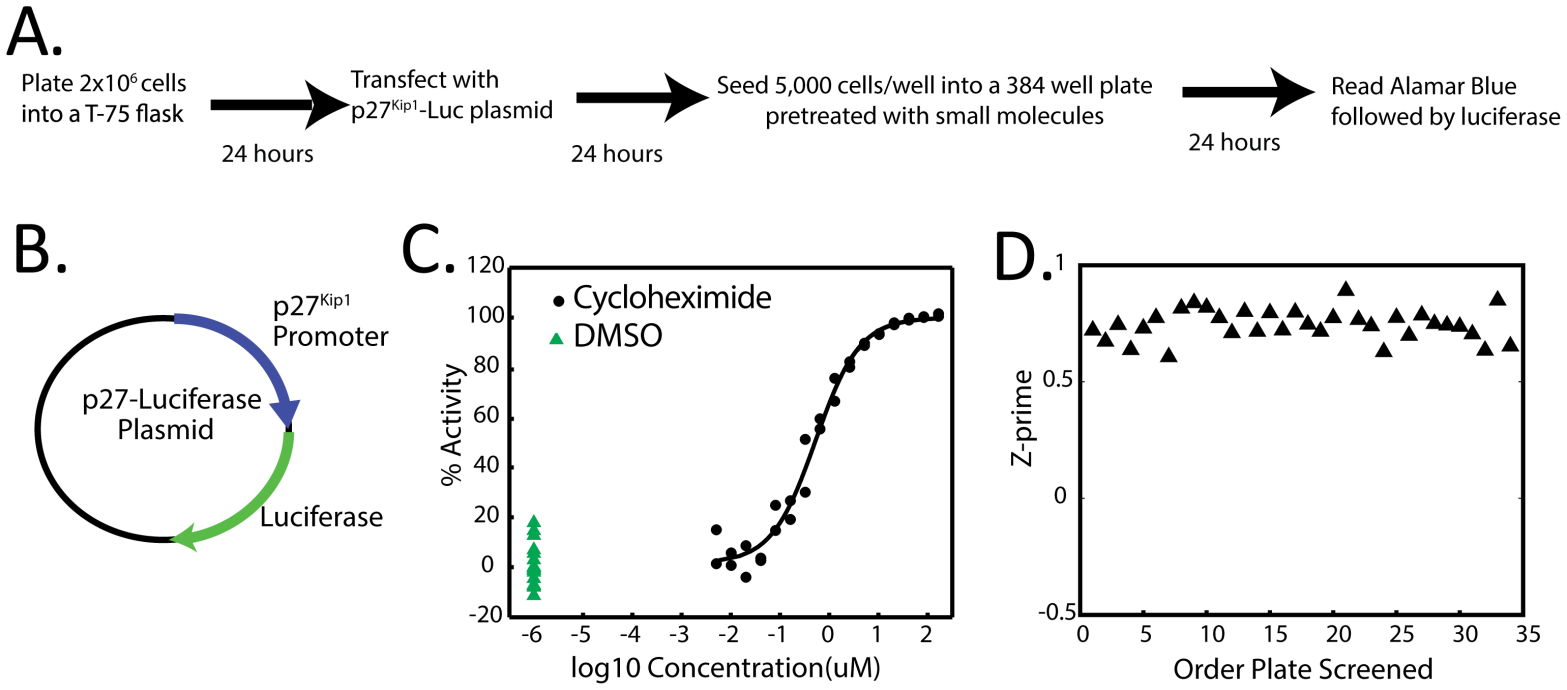
Design and validation of a high throughput screen to antagonize *p27^Kip1^* transcription in HeLa cells. A. Workflow for the primary screen. B. Schematic of the 4-kb human *p27^Kip1^* promoter driven luciferase plasmid used in this study. C. Performance of the *p27^Kip1^*-luciferse assay against negative controls (DMSO, green triangle) and titrating amounts of the positive control (cyclohexamide, black circle). D. Z’ factor calculations for assay performance over 34 plates from the primary screen. Mean Z’ factor = 0.74±0.06.

Luminescence of LacZ was measured using the duel light luminescence kit from Life Technologies (#T1003) following the workflow diagrammed in Fig. 1D, and the same conditions optimized for p27^Kip1^-luciferase transfections. Luminescence was quantified on either a GloMax Multi+ (Promega) or the Envision HTS microplate reader.

### mRNA Isolation

Cells were grown to ∼ 50% confluence at which time A2CE was added. 16–24 hours later cells were harvested in RNA stat 60 (Tel-Test, #CS-112) and mRNA harvested following the manufacturer’s protocol. The resulting mRNA was quantified (Thermo, NanoDrop 2000), and further used to generate cDNA (Life Technologies, #4368813). qPCR was performed on 2ng of cDNA run in multiplex against human p27^Kip1^ (Life Tech, Hs01597588), or mouse p27^Kip1^ (Life Tech, Mm00438168), combined with 18S (Life Tech, 4310893E) as the internal control.

### Cochlear Explants

Organs of Corti were harvested from P1-P4 mice, plated on Matrigel (BD bioscience, #356234), and grown in DMEM (supplemented with 1% FBS, 10 μg/μL ciprofloxin, at 37°C, 5% CO_2_, 95% relative humidity). A2CE (250 nM-10 μM) was added after 24 hours, and explants were harvested after additional 24 hour incubation. Isolation and quantification of the mRNA was done the same as above.

### Immunoblot

HeLa cells were harvested and homogenized in RIPA buffer (10 mM Tris (pH 7.4), 100 mM NaCl, 1 mM EDTA, 1 mM EGTA, 0.1%SDS, 1% Triton-X100, 0.5% sodium deoxycholate, 10% glycerol, + protease and phosphatase inhibitors) and total protein was quantitated using a BCA assay (Thermo scientific, #23225). Samples were boiled for 5 min with SDS sample loading buffer prior to loading. Samples were run on 4–20% precast polyacrylamide gels (Bio-Rad, 456–1096) after which they were transferred to a polyvinylidene difluoride membranes (Millipore, #IPHVH07850). Membranes were blocked in 5% (w/v) fat-free milk–PBST (phosphate buffer with 0.05% Tween 20) for 1 hour at room temperature after which the membranes were incubated with rabbit anti-p27^Kip1^ polyclonal antibody (#06-445, Upstate) or anti-β-actin antibody (#3700, cell signaling) in PBST overnight at 4°C. The membrane was washed three times with PBST followed by incubation in HRP-anti-mouse IgG or HRP-anti-rabbit IgG secondary antibodies diluted 1∶5000 in PBST for 1 hour at room temperature. After washing three times with PBST, the specific bands were developed on the films via using SuperSignal West Pico Chemiluminescent Substrate (Thermo Scientific). Densitometry was performed using ImageJ (U.S. National Institutes of Health).

### Isolation of Full Length p27^Kip1^

Full-length p27^Kip1^ protein was expressed in *Escherichia Coli* as 6xHis fusion constructs. The protein was purified by nickel affinity chromatography and then digested with thrombin to remove the fusion tag. Further purification by reverse-phase high performance liquid chromatography (HPLC) using a C4 column (Vydac) and 0.1% trifluoroacetic acid-containing water/acetonitrile solvent system yielded high purity protein. Protein concentration was determined by UV absorbance at 280 nm using a molar extinction coefficient of 15,470 M^−1^*cm^−1^.

### Statistics

Statistics were performed using OriginLab 8.5. Where applicable, one-way ANOVAs with Bonferroni were run to test for mean difference, or a two sample T-test if only two conditions were compared.

## Results

### Creation and Validation of p27^Kip1^-luciferase Assay for High Throughput Screening

We optimized our transfection and screening procedure (diagrammed in Fig. 1A) using a *p27^Kip1^*-luciferase plasmid transfected into HeLa cells. We have previously used the p27^Kip1^ plasmid [12], but the backbone was not suitable for high throughput screening (HTS). We cloned the 4-kb human *p27^Kip1^* promoter into the BamHI site of the pGL4.17 commercially available luciferase backbone (Fig. 1B). To ensure a robust assay, we chose a positive control in each plate that should maximally repress the luciferase signal, and thus chose the generalized translation inhibitor cycloheximide. Titration of cyclohexamide produced optimal repression of luciferase at 200 μM and was then defined as 100% luciferase inhibition (Fig. 1B). Z’ factor (measure of robustness of a HTS) was calculated for each plate run in the HTS and is displayed in Fig. 1C. The overall mean of the Z’ factor from our assay was 0.74±0.06, demonstrating our assay is well suited for HTS. Cell viability was monitored during our HTS, since inhibition of luciferase could be due to cell death and not repression of *p27^Kip1^*. We chose to use alamar blue, an oxidation-reduction indicator, which is converted to a fluorescent form proportionally to cellular metabolism (Fig. 1D) and is commonly used in HTS as a measure of cell viability.

### High Throughput Screening of the Combined FDA/bioactive Libraries Reveals 111 Primary Hits

**Figure 2:**
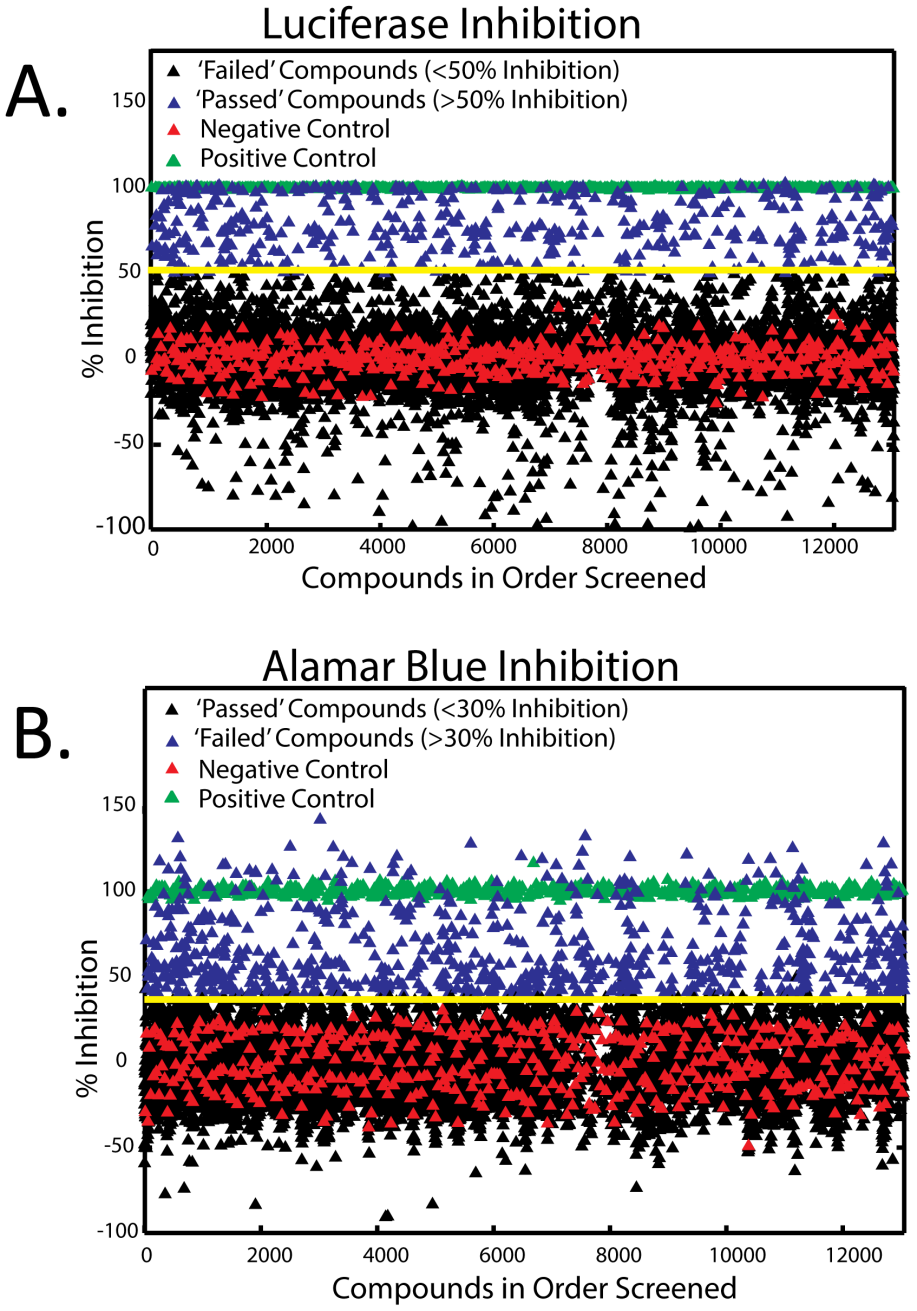
Primary screening for inhibition of p27^Kip1^-luciferase. A. Percentage of luciferase inhibition by each compound screened (12 μM), compared to positive control cycloheximide (green), and negative control DMSO (red). Arbitrary threshold was set at 50% luciferase inhibition (yellow line). Compounds which “passed” (blue) and “failed” (black) are shown. B. Percentage of alamar blue inhibition by each compound screened (12 μM) compared to positive control cyclohexamide (green), and negative control DMSO (red). Arbitrary threshold was set at 30% alamar blue inhibition (yellow line). Compounds which inhibited alamar blue more than 30% “failed” (blue), and less than 30% “passed” (black).

Once we validated the primary assay, we proceeded to the primary screen of the “bioactive” library. Small molecules were added to a final concentration of 12 μM (following the workflow from Fig. 1A). Alamar blue signal was measured first for cell viability, followed by luciferase activity. Any compound which demonstrated at least 50% luciferase inhibition (Fig. 2A, when compared to 200 μM cycloheximide) but less than a 30% drop in cell viability (Fig. 2B) were considered primary hits, and passed on to the first of the secondary screens. We obtained 111 primary hits, representing ∼2.5% hit rate.

### Secondary Screening Validated Four p27^Kip1^ Transcriptional Inhibitors

**Figure 3:**
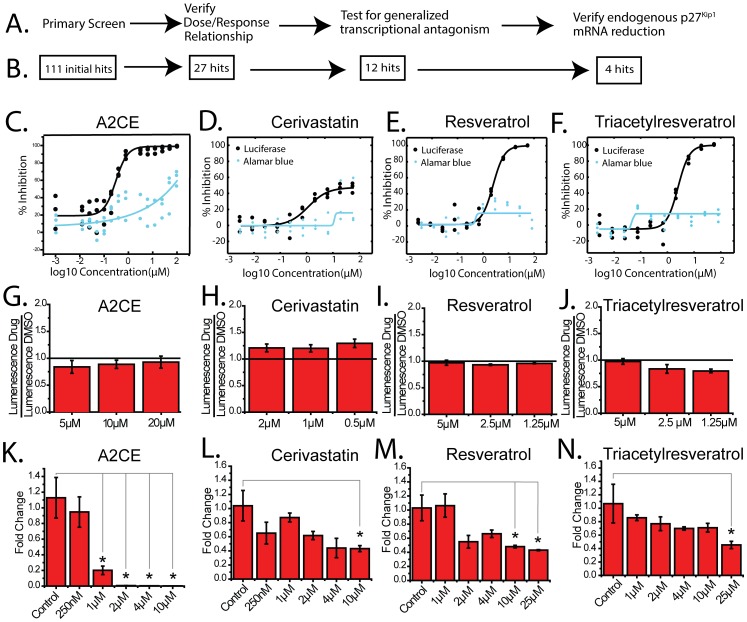
Secondary screening of primary hits reveals four true *p27^Kip1^* inhibitors. A. Workflow of the secondary screening procedures. B. Remaining hits after each step of the secondary screen. C–F. Dose-dependent luciferase inhibition (black) and alamar blue inhibition (blue) of each compound (n = 3, Table 1 lists the calculated IC_50_’s). Curves were fitted by a sigmoidal dose-response curve. G–J. Luciferase levels (normalized to DMSO) over 3 doses of each compound showing no effect on SV-40 driven luciferase (n = 3). Green line represents no change from vehicle DMSO to A2CE. K–N. Dose-dependent decrease in endogenous p27^Kip1^ mRNA of each compound normalized to that in DMSO control (RT-qPCR) in HeLa cells (n = 3). Mean ± S.E.M., * p<0.05 (One way ANOVA, followed by bonferroni for means comparison).

Primary hits from HTS may contain many “false-positives” ranging from toxic compounds to naturally fluorescence-quenching molecules. To help remove these false-positive hits from our list, we designed three progressively more restrictive secondary screens (Fig. 3A). First to confirm the effect of these compounds on inhibiting the activity of the *p27^Kip1^*-luciferase reporter, we tested all primary hits in a dose response analysis and assayed not only the luciferase response for *p27^Kip1^* inhibition, but also the alamar blue levels for cell survival. IC_50_ values were calculated from the luciferase response and are displayed for each final compound in Table 1. Only compounds that demonstrated a robust dose response relationship with the luciferase values, and no noticeable effect on alamar blue over the effective doses (Fig. 3C–F) were passed to the next secondary screen.

**Table 1:** Four p27^Kip1^ inhibitors and calculated IC_50_ values that passed all primary and secondary screens.

Name	Calculated IC_50_ (uM)	95% Confidence Interval
**ALSTERPAULLONE, 2-CYNANOETHYL**	0.2	–0.129, 0.530
**CERIVASTATIN SODIUM**	1	0.5, 2.0
**RESVERATROL**	2.49	2.10, 2.94
**TRIACETYLRESVERATROL**	2.5	1.9, 3.3

The activity data for individual compounds were fit into sigmoidal dose-response curves (n = 3 per compound, Fig. 3C–F) to derive IC_50_ values.

Since it is possible that any positive hits obtained from the primary screen could represent generalized transcriptional inhibitors that are non-specific to *p27^Kip1^*, we used an independent plasmid utilizing the SV40 promoter to drive LacZ expression (Fig. 3G–J). LacZ expression was measured in a luminescent assay over 3 concentration points of the compound, and any reduction in luminescence would reflect a generalized transcriptional repression, and would eliminate the compound.

After the first two phases of secondary screens, we asked if the addition of the remaining compounds could repress endogenous *p27^Kip1^* expression (measured by quantitative real time PCR) of HeLa cells instead of the transient transfected reporter plasmid (Fig. 3K–N). Table 1 describes the full list of compounds that passed both the primary and secondary screens as well as calculated IC_50_ values.

### Alsterpaullone, 2-cyanoethyl Treatment Reduces p27^Kip1^ Protein Levels

**Figure 4:**
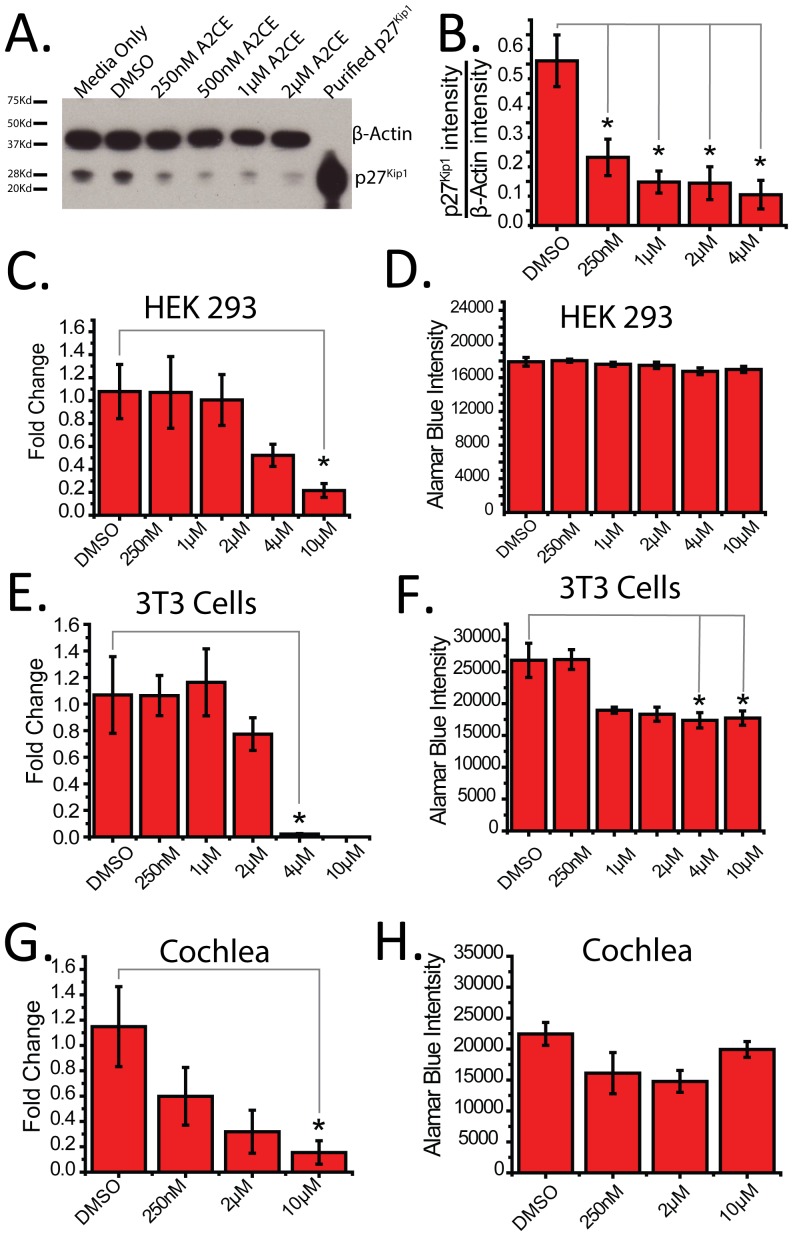
Alsterpaullone, 2-Cyanoethyl inhibits *p27^Kip1^* transcription from diverse cellular origins, and reduces p27^Kip1^ protein levels. A. Immunoblot of p27^Kip1^ and β-actin with indicated concentration of A2CE in HeLa cells. B. Relative intensity of p27^Kip1^ normalized to β-actin by densitometry (n = 3) with indicated concentrations of A2CE. C. Fold changes of endogenous p27^Kip1^ mRNA (normalized to 18s) in HEK cells treated with indicated concentrations of A2CE normalized to DMSO (n = 3). D. Alamar blue cell viability assay in HEK cells treated with indicated concentrations of A2CE. E. Fold changes of endogenous p27^Kip1^ mRNA (normalized to 18s) in 3T3 cells treated with indicated concentrations of A2CE normalized to DMSO (n = 3). F. Alamar blue cell viability assay in 3T3 cells treated with indicated concentrations of A2CE G. Fold changes of endogenous p27^Kip1^ mRNA (normalized to 18s) in cochlear explants treated with indicated concentrations of A2CE normalized to DMSO (n = 3). H. Alamar blue cell viability assay in cochlear explants treated with indicated concentrations of A2CE. Mean ± S.E.M., * p<0.05 (One way ANOVA, followed by bonferroni for means comparison).

After validation of these four compounds as *p27^Kip1^* transcription antagonists, we focused our attention on A2CE because the other three compounds demonstrated only a modest reduction on the level of *p27^Kip1^* mRNA. First, we wanted to ensure that A2CE treatment resulted in a reduction of endogenous p27^Kip1^ at the protein level in HeLa cells. To understand this, we assayed p27^Kip1^ protein levels using an immunoblot procedure. We added progressively higher doses of A2CE, and observed an immune positive band at 28 kD when probed with p27^Kip1^ specific antisera that decreased in intensity with higher treatment concentrations of A2CE (Fig. 4A). β-actin was used as a loading control. Band intensities were quantified using densitometry (Fig. 4B) and displayed as average p27^Kip1^ intensity normalized to β-actin intensity. These results confirmed that A2CE’s inhibitory effects on *p27^Kip1^* transcription eventually led to a reduction at the protein level.

### Alsterpaullone, 2-cyanoethyl Treatment Reduces p27^Kip1^ mRNA across Diverse Cell Lines and in Cochlear Explants

To understand whether addition of A2CE was only relevant in HeLa cells, we treated both HEK-293 and 3T3 cells with varying doses of A2CE and assayed the abundance of *p27^Kip1^* transcript after 24 hours of treatment. Significantly less *p27^Kip1^* mRNA was detected in both HEK-293 and 3T3 cells when assayed by quantitative PCR (qPCR) normalized to the internal control, 18s (Fig. 4C, E). To understand if these cell lines were more sensitive to A2CE than HeLa cells, we also measured cell viability by alamar blue, and detected a small but statistically significant reduction in viability at the highest doses in 3T3 cells but not in HEK293 cells indicating that 3T3 cells are slightly more sensitive to A2CE than HeLa cells. Finally, we wanted to understand the effects of A2CE treatment on cochlear explants, one of the tissue types where repression of *p27^Kip1^* may be beneficial. Postnatal (P) 1-P4 cochleae were isolated and plated in matrigel. After 1 day of recovery the explants were treated with varying doses of A2CE. The following day whole cochlear mRNA was harvested, or whole explants were treated for alamar blue for viability. Fig. 4G demonstrates a significant reduction of *p27^Kip1^* mRNA after treatment with 10 μM A2CE, with no significant changes in viability (Fig. 4H).

### Alsterpaullone, 2-cyanoethyl inhibits FoxO3a Binding to the p27^Kip1^ Promoter

**Figure 5:**
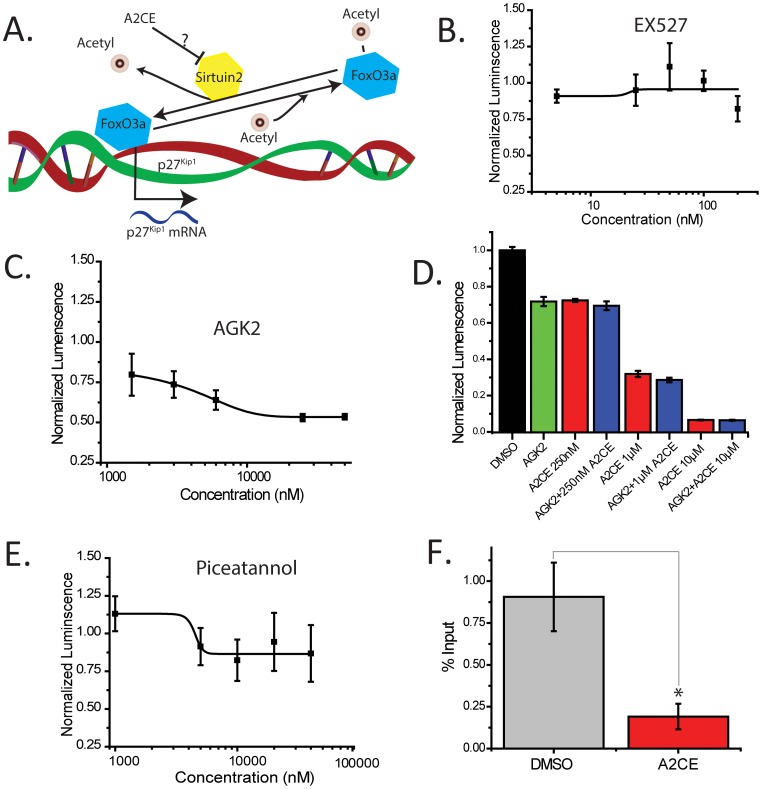
Alsterpaullone, 2-Cyanoethyl prevents FoxO3a from binding to the *p27^Kip1^* promoter. A. Proposed schematic on how FoxO3a interacts with the *p27^Kip1^* promoter, and where A2CE is hypothesized to interact with it. B–E. *p27^Kip1^*-luciferase levels (normalized to alamar blue levels) with indicated doses of the Sirtuin 1 inhibitor EX527 (B, IC_50_ = 100 nM, n = 3), the Sirtuin 2 inhibitor AGK2 (C, IC_50_ = 3.5 μM, n = 3). Values were fit to a sigmoidal dose-response curve. D. Combination of A2CE (250 nM–10 μM, n = 3 for each dose) and AGK2 (10 μM, n = 3) had no synergistic effect on luciferase intensity when compared to A2CE alone (red, n = 3) or AGK2 alone (green, n = 3). All values are normalized to the DMSO control (black, n = 3). E. *p27^Kip1^*-luciferase levels (normalized to alamar blue levels) with indicated doses of the resveratrol metabolite piceatenol (E, n = 3). Values were fit to a sigmoidal dose-response curve. F. Percentages of input using chromatin immunoprecipitation (ChIP) for FoxO3a followed by quantitative real-time PCR for the FoxO3a binding site within the p27^Kip1^ promoter with DMSO or 10 μM A2CE (n = 3). Mean ± S.E.M., * p<0.05 (Students T-test).

Although A2CE has no known effects on p27^Kip1^ levels, other types of paullones can inhibit certain types of NAD+ dependent deacetylases, primarily the Sirtuins [32]. The Sirtuins deacetylate FoxO3a and regulate *p27^Kip1^* transcription (Fig. 5A). To ascertain if Sirtuin inhibition could phenocopy the A2CE inhibition in the *p27^Kip1^* luciferase assay, we exposed our *p27^Kip1^*-luciferase assay to different doses of a known Sirtuin 1 (Sirt1) inhibitor (EX527, IC_50_ = 100nM, Fig. 5B), and a known Sirt2 inhibitor (AGK2, IC_50_ = 3.5 μM, Fig. 5C) in HeLa cells. Treatment with the Sirt2-specific inhibitor phenocopied the results with A2CE, while the addition of the Sirt1-specific inhibitor had no effect, suggesting that Sirt2 is responsible for A2CE effects on *p27^Kip1^* transcription in our assay. To support the idea that A2CE and AGK2 function in the same pathway we combined both AGK2 and A2CE and asked if these two compounds acted synergistically to repress luciferase levels. In Fig. 5D we treated HeLa cells transfected with the p27^Kip1^-luciferase plasmid to AGK2 (10 μM), and A2CE (250 nM-10 μM) and did not observe any synergistic effect on luciferase luminescence at any dose. This implies that AGK2 and A2CE function in the same pathway.

Resveratrol is a known Sirtuin agonist, and surprisingly, it was also one of the hits from our screen (Table 1).To determine whether promoting protein decetylation also repressed *p27^Kip1^*, we treated HeLa cells transfected with the *p27^Kip1^-luciferase* plasmid with piceatenol, a metabolite of resveratrol [31] that promotes protein deacetylation [32]. Piceatenol did not phenocopy the inhibition of *p27^Kip1^*-luciferase by resveratrol (Fig. 5D), suggesting that resveratrol is inhibiting *p27^Kip1^* through a different mechanism.

Finally, we examined the downstream effects of Sirt2 antagonism (diagrammed in Fig. 5A) by assaying FoxO3a binding to the *p27^Kip1^* promoter after 10 μM A2CE treatment by chromatin immuno-precipitation (ChIP), followed by qPCR. We observed a significant decrease in FoxO3a binding to the *p27^Kip1^* promoter upon addition of 10 μm A2CE (Fig. 5E), demonstrating that *p27^Kip1^* transcriptional repression by A2CE is through the lack of FoxO3a binding.

## Discussion

We successfully performed and validated a high-throughput screen for *p27^Kip1^* transcriptional inhibition, and conducted a battery of secondary screens to validate our hits. From the “bioactive” library, we obtained 111 initial hits, which were reduced down to four true *p27^Kip1^* transcription inhibitors. Because the *p27^Kip1^* screen itself was not biased for inhibitors or activators of *p27^Kip1^*, our screen also identified over a hundred primary hits for compounds, which promote *p27^Kip1^* transcription. These compounds may help minimize growth of tumors that have lost *p27^Kip1^* expression and are worth investigating in future studies.

Following the screen, we focused on understanding how our lead hit, A2CE, repressed *p27^Kip1^* transcription and discovered a novel function for this compound. Although not FDA-approved, A2CE has been classified as an anti-tumor compound due to its ability to inhibit CDK’s directly, arresting cells in the G1/S phase of the cell cycle. Our work may therefore be useful in chemotherapy against certain cancers. A2CE could be especially effective in cancers that exhibit p27^Kip1^ mislocalization causing enhanced metastasis [21], [22]. A2CE would not only inhibit cell growth by inhibiting CDK directly, but also prevent metastasis by repressing cytoplasmic p27^Kip1^. Our data provide evidence for a novel use for A2CE on preventing cancer metastasis that warrants further investigation. Inhibition of p27^Kip1^ without proliferation is still beneficial for regenerative therapy. Use of A2CE to promote stem cell pluripotency [9] and transdifferentiation [33] may still prove fruitful, and warrants further studies. If successful, A2CE offers a potential benefit over compounds which only promote proliferation, because A2CE should not have tumorigenic side effects associated with some proliferative therapy.

Paullones are a wide and diverse class of compounds, which act as ATP mimetics and were first described as inhibitors for various CDKs [34]. It is well known that CDKs (primarily CDK2, 4, and 6) hyperphosphorylate the retinoblastoma protein (pRB) causing the release of the E2F family of transcription factors and further promote transcription of their target genes [35]. One of the target genes of E2F1 is *p27^Kip1^* [24], implying that inhibition of the CDKs could explain the observed decrease in *p27^Kip1^* transcription. Although possible, it is unlikely to be a major mechanism for repressed *p27^Kip1^* transcription in our assays. First, the IC_50_ of A2CE against CDK2 is an order of magnitude more potent than the IC_50_ determined from our assay, implying that an even more potent IC_50_ would have been expected. Second, kenpaullone, an effective inhibitor of CDKs [36] is included in our library and did not pass our primary screen. Third, there are over 100 CDK2 inhibitors, and over 250 various CDK inhibitors contained within our library. If inhibition of CDKs were the primary method to reduce *p27^Kip1^* transcription in our assay, we would have expected these compounds to be more enriched than was observed. It is still possible that some amount of the *p27^Kip1^* repression is due, at least in part, to the lack of E2F1 transactivation via loss of pRB phosphorylation by CDKs.

The paullone analogs are all characterized as inhibitors of CDKs, and interestingly some are even used in the production of induced pluripotent stem cells [37]. Thus, finding mechanisms where Kenpaullone and A2CE, two highly similar compounds, can result in radically different phenotypes had been problematic. A recent study by Trapp et al. helped clarify this issue by demonstrating that some members of the paullone class could display NAD+ mimetic behavior, and since the Sirtuin class of deacetylases require NAD+, some paullones may be able to inhibit the Sirtuins [32]. Trapp et al. demonstrated that kenpaullone did not have any Sirtuin inhibitory activity, but some modifications (benzylation of the lactam nitrogen, or the introduction of a hydroxyamidine structure) resulted in a very low level of Sirtuin inhibition (IC_50_ near 50 μM). This observation delineated the activity of kenpaullone from other members of this family of CDK inhibitors, and further supports the idea that A2CE can affect *p27^Kip1^* transcription through Sirtuin inhibition, albeit by >2 orders of magnitude higher affinity than the modified paullones made in that study.

Sirt1 and Sirt2 have both been described as affecting FoxO3a acetylation status [25], [38], so it is unclear why only Sirt2 inhibitors demonstrate *p27^Kip1^* inhibition in this study. This could be due to Sirt2 playing a larger role in FoxO3a deacetylation in our system. Alternatively, since our primary screening procedure utilized transient transfection of HeLa cells, the primary location of our plasmid should be cytoplasmic. Since Sirt1 is described as primarily cytoplasmic, any deacetylation done by Sirt1 may not be captured in our primary screen. On face value, this may also raise the possibility that some of our primary hits are artificial, since we screened for inhibitors of plasmid based *p27^Kip1^* expression. However, it is important to remember that our final secondary screen did not utilize plasmids but instead looked at endogenous *p27^Kip1^* expression, demonstrating that all four of the hits we identified are not specific to plasmid based transcription, but represent true *p27^Kip1^* inhibitors.

Use of luciferase-based assays for transcriptional screening has been around for over a decade, but to our knowledge, using this method to screen for small molecules that transcriptionally repress CDK inhibitors had not been attempted. We successfully screened our “bioactive” library and discovered four novel compounds which repress *p27^Kip1^* transcription. Our most potent hit, A2CE, yielded affinities in the sub-micro molar range, which encouraged us to identify the molecular mechanism through which A2CE repressed *p27^Kip1^* transcription. We demonstrate that this inhibition occurs mainly through repression of FoxO3a binding to the *p27^Kip1^* promoter. We further plan to characterize the effects of A2CE in regenerative systems, such as the inner ear, though local delivery, given our results in cochlear explant culture. Furthermore, since we have verified the ability of our assay to detect *p27^Kip1^* inhibitors, we can pursue other larger unbiased libraries, or smaller specialized libraries focused on FoxO3A regulation, to further identify compounds which may yield p27^Kip1^ repressors with higher affinities. We are hopeful that our discoveries of *p27^Kip1^* inhibitors will not only be useful for tissue regeneration, but that the assay itself may yield more potent regulators of *p27^Kip1^*.
